# Validation of a *pan*-orthopox real-time PCR assay for the detection and quantification of viral genomes from nonhuman primate blood

**DOI:** 10.1186/s12985-017-0880-8

**Published:** 2017-11-03

**Authors:** Eric M. Mucker, Christopher Hartmann, Donna Hering, Wendy Giles, David Miller, Robert Fisher, John Huggins

**Affiliations:** 1 Virology Division, U.S. Army Medical Research Institute of Infectious Diseases, 1425 Porter Street, Fort Detrick, Frederick, MD 21702-5011 USA; 2 Quality Assurance & Regulatory Compliance Office, U.S. Army Medical Research Institute of Infectious Diseases, 1425 Porter Street, Fort Detrick, Frederick, MD 21702-5011 USA; 3 Office of Regulated Studies, U.S. Army Medical Research Institute of Infectious Diseases, 1425 Porter Street, Fort Detrick, Frederick, MD 21702-5011 USA; 4 Division of Medicine, U.S. Army Medical Research Institute of Infectious Diseases, 1425 Porter Street, Fort Detrick, Frederick, MD 21702-5011 USA

## Abstract

**Background:**

In 1980, smallpox disease was eradicated from nature and *Variola virus*, the etiological agent of smallpox, was confined to two laboratories, one located in Russia (Moscow) later moved to VECTOR (Novosibirsk, Siberia) and one in the United States (CDC Atlanta). Vaccinations among the general public ceased shortly after the successful eradication campaign, resulting in an increasingly immunologically susceptible population. Because of the possibility of intentional reintroduction of *Variola virus* and the emergence of other pathogenic poxviruses, there is a great need for the development of medical countermeasures to treat poxvirus disease. It is highly likely that the U.S. FDA “animal rule” will be necessary for regulatory approval of these interventions. Therefore, relevant animal models and the associated supporting assays will require development to stand up to regulatory scrutiny.

**Methods:**

An optimized real time PCR assay for the detection of orthopoxviruses has been developed by researchers at the United States Army Research Institute of Infectious Diseases (USAMRIID). To support animal studies that will be used to support approval of medical countermeasures by the U.S. FDA, the assay was designed to quantitate poxvirus genomic DNA in a nonhuman primate (cynomolgus macaque) blood matrix as a measurement of viremia. This manuscript describes the validation of the process, including DNA extraction from whole blood anticoagulated with EDTA, for obtaining and quantitating monkeypox genomes by evaluating precision, accuracy, the standard curve, specificity, robustness and stability of the assay and/or components of the assay.

**Results:**

The assay had a lower limit of quantitation of 50 genome copies/5 uL sample, upper limit of quantitation of 5 × 10^7^ GC/5uL sample and a limit of detection of 2.5 genome copies /5uL sample. The assay was specific for orthopoxvirus. Matrix effects were detected and suggest the presence of PCR inhibitor(s) that was co-extracted with the target DNA.

**Conclusions:**

The assay has been validated for the purpose of quantitating monkeypox viral load in blood from cynomolgus macaques. This assay has and will continue to support submissions to the FDA for approval of antiviral therapeutics for smallpox.

**Electronic supplementary material:**

The online version of this article (10.1186/s12985-017-0880-8) contains supplementary material, which is available to authorized users.

## Background

The cynomolgus macaque/intravenous *Monkeypox virus* model was developed to parallel certain disease aspects of smallpox [1–3]. More specifically, the model is thought to recapitulate the events following secondary viremia in human smallpox patients [2]. After intravenous administration of the virus, the animals develop fever, viremia, a progressive and centrifugal rash, and exhibit dose-dependent morbidity and mortality. Correlates of protection, such as viremia, can be used for approval of new drugs under the FDA’s accelerated approval mechanism, such as the influenza vaccines Fluad and Flucelvax. Quantitative viral titer measurements like plaque assay method(s) and polymerase chain reaction (PCR) analysis by agarose gel electrophoresis have given way to sensitive and rapid PCR methods using minor groove binding (MGB) probe technology to detect amplified viral nucleic acids. The Diagnostic Systems Division (DSD), at the United States Army Research Institute of Infectious Diseases (USAMRIID), used MGB chemistry to develop and optimize an assay for the diagnostic detection of orthopox species [4]. This assay specifically detects and quantifies an orthopox specific target within the viral hemagglutinin (HA) gene sequence using the Roche LightCycler® (Indianapolis, IN). We subsequently adapted this platform to assess disease progression and for the evaluation of potential antiviral candidates. For the assay to be truly useful in support testing and evaluation work to support medical countermeasure development, it needed to be validated to the extent that it would be acceptable by the appropriate regulatory body (in this case, the U.S. FDA).

The goal of this validation process was to demonstrate the overall reliability and robustness of a well optimized assay. The validation sought to determine the performance characteristics of a real time PCR assay for the *pan*-Orthopox virus HA gene in cynomolgus macaque blood. More specifically, we assessed: repeatability; intermediate precision; accuracy of the combined extraction and amplification method; upper and lower limits of the standard curve; storage stability of the HA standard (Variola Bangladesh HA gene), positive extraction control (PEC), and MGB master mix; specificity of the method using other orthopox viruses and non-orthopox viruses; and robustness of the method using different lots of reagents and different light cycler instruments. This validation was conducted in accordance with the FDA Good Laboratory Practice (GLP, 21 CFR Part 58) [5].

## Methods

Because the validation was conducted in compliance with 21CFR part 58, a study protocol, standard operating procedures and other documentation were developed and implemented before the onset of the study. Deviations to any of the aforementioned documents were documented and formally reported. The data packet, including documentation, and final report were reviewed and accepted by USAMRIID’s Quality Assurance and Regulatory Compliance Office (QARCO). For simplicity, the nomenclature for lot numbers, test and control samples, study assays, and study runs have been re-designated.

### Test articles and reference materials

*Monkeypox virus* strain Zaire 79, was provided by Dr. Joe Esposito (CDC) and extracted DNA from *Human alphaherpes virus 1* strain KOS (referred to as *Herpes simplex virus 1 or* HSV-1), *Human alphaherpes virus 2* strain 186 (referred to as *Herpes simplex virus 2 or* HSV-2), *Camelpox virus* strain Somalia, *Cowpox virus* strain Brighton, *Rabbitpox virus* strain Utrect, *Vaccinia virus* strain Copenhagen, and *Variola virus* strain Bangledash were provided by the USAMRIID Virology and Diagnostic Systems Divisions. Unpurified viral stocks were utilized in these studies.

### DNA extractions

Test and control samples were extracted using the Qiagen QIAamp DNA Mini kit (catalog# 51306) adapting the manufacturer’s instructions. Briefly, pre-extraction inactivation was performed by adding 100uL of sample to a tube containing 100uL of PBS, 200uL of Qiagen Buffer AL, and 20uL of proteinase K. The tubes were placed in a 56° ± 2 °C waterbath for 60 min to inactivate the virus (data not shown). Extraction proceeded according to manufacturer’s directions and samples were eluted in 100uL volume. Each extraction was designated by a letter given in alphabetical order and will be referred to as such.

### Test and control samples

Orthopox negative EDTA blood was collected from a single, orthopox naïve cynomologus macaque (*Macaca fascicularis*), aliquoted and stored at −70° ± 10 °C for use as negative control (NC) samples. The positive extraction control (PEC) was prepared by spiking the NC with 1 × 10^5^ pfu/ml of *Monkeypox virus* and stored at −70° ± 10 °C. Whole blood, collected from NHPs from previous in vivo monkeypox studies were stored at −70 °C ± 10 °C, extracted and stored at −20 °C ± 10 °C, or short term at 2–8 °C. Orthopoxvirus positive test samples (PTS) were either extracted orthopox positive NHP blood (from study) or orthopoxvirus negative NHP blood (from study or naïve animal) spiked with either stock *Monkeypox virus* or extracted viral DNA. The PTS used from study material were post infection day 2 (1.6 × 10^1^ GC/5ul), day 5 (5.6 × 10^3^ GC/5ul), and day 8 (2.2 × 10^5^ GC/5ul) and were all from the same animal.

### Standard curve

A preparation of the HA insert from a plasmid containing the *Variola virus* strain Bangladesh (VARV-BSH) HA gene was used as the standard (HA Standard) for quantifying viral DNA as genome copies (GC). The HA (J7R) gene of *Variola virus* strain Bangladesh (GenBank accession number L22579) was cloned [4], propagated in bacterial cultures, and the plasmids were extracted using a commercial kit (Qiagen). The insert was cut from the plasmid using EcoR1 restriction enzyme and confirmed by electrophoresis. The band was excised and purified from the gel using Qiagen’s “QIAquick Gel Extraction” Kit and the manufacturers protocol (QIAquick Gel Extraction Kit using a Microcentrifuge). The excised band was subsequently quantitated on a Beckman DU series 500 spectrophotometer. The DNA was diluted in log increments from 1 × 10^6^ genome copies/uL to 5 genome copies/uL unless otherwise stated.

### Pan-Orthopox assay

The *pan*-orthopox PCR assay has been previously described by [4], with the exception of quantitation strategy utilizing the aforementioned standard curve. Each LightCycler reaction was comprised of 5.0 μl of DNA sample and 15 μL of Master Mix, containing 0.5 mM OPSP-F89 (primer sequence: 5′ - GAT GAT GCA ACT CTA TCA TGT A - 3′), 0.5 mM OPSP-R219 (primer sequence: 5′ - GTA TAA TTA TCA AAA TAC AAG ACG TC - 3′), 0.1 mM OPX-P143S-MGB (probe sequence: 6’FAM - AGT GCT TGG TAT AAG GAG - MGBNFQ - 3′), 1X dNTPs, and 1X Buffer (Idaho Technologies), in molecular biology grade water. MGB Master Mix was stored at −20 °C ± 10 °C prior to use. All assays were performed on a single LightCycler® (Roche) with the exception of the robustness testing where two separate units were utilized. Data was captured on a LightCycler equipped with Software version 3.5.3; because of the concise data reporting features and expanded data analysis capabilities afforded by the v4.0 software, results were analyzed and reported using this version.

### Calculations and statistical analyses

The LightCycler® v4.0 software performed an Absolute Quantification analysis by plotting the CTs (crossing threshold or crossing point) of Test Samples against the concentrations (GC values) and CTs of the standards. The X axis of the standard curve represented the log of the initial concentration of DNA and the Y axis represented the CT. CT and GC values were transferred to Excel Microsoft (2003) spreadsheets. GC values were used for the calculation of means, standard deviation (SD), coefficient of variance (CV) and recovery. Acceptable CV for mean duplicate values and the range of standards, controls, and efficiency values were established prior to the validation so erroneous results due to technician error could be dropped (Table 1). This acceptance criterion was used for the mean duplicate values from each run as well as for the mean values for each assay (usually comprised of 3 runs). Per the study protocol, a single Standard Curve value could be dropped when it did not meet the acceptance criteria and the curve recalculated.

**Table 1:** Acceptance criteria for standards, controls, and test samples

Standards & Controls	Acceptable Range (GC/5 μl)Low to High		Acceptable %CV
PEC	1.58 × 10^3^	1.58 × 10^4^	≤ 40%
50 HA Standard	1.58 × 10^1^	1.58 × 10^2^	≤ 40%
500 HA Standard	1.58 × 10^2^	1.58 × 10^3^	≤ 30%
5000 HA Standard	1.58 × 10^3^	1.58 × 10^4^	≤ 30%
50,000 HA Standard	1.58 × 10^4^	1.58 × 10^5^	≤ 30%
500,000 HA Standard	1.58 × 10^5^	1.58 × 10^6^	≤ 30%
5,000,000 HA Standard	1.58 × 10^6^	1.58 × 10^7^	≤ 30%
PTS	NA	NA	≤ 30%
Efficiency	1.8	2.2	< 10%

*PEC* positive extraction control

*PTS* positive test sample

## Results

### Verification of precision

According to FDA guidance, “Precision describes the closeness of individual measures of a an analyte when the procedure is applied repeatedly to multiple aliquots of a single homogenous volume of biological matrix” [6]. In our study, repeatability and intermediate precision were assessed by determining the %CV for orthopox gene copies (GC) for all pertinent sample types (standard curve: HA gene; Controls: Positive and negative extraction controls, and Test Samples) for each assay. Based on the assays intended use (comparing multiple log differences of viremia in vivo) a %CV of less than 30% was deemed acceptable for repeatability/intermediate precision comparisons. Each assay consisted of 3 LightCycler runs. Two sets (A and B) of 3 assays were completed for a total of 9 runs per set (Table 2). Each set of assays included two analysts. Assays 1, 2, and 3 (from set A and B) were used to determine repeatability, assay 1 and 3 (from set A and B) were used to determine precision of samples run on different days by the same analyst, and assay 1 and 2 (from set A and B) were used to determine precision of samples run on different days by different analyst.

**Table 2:** Precision testing for repeatability and intermediate precision

Set	Assay	Run	Analyst	Day	Repeatability	Same analyst	Different analyst
A	1	01, 02, 03	1	1	X	X	X
A	2	04, 05, 06	2	2	X		X
A	3	07, 08, 09	1	3	X	X	
B	1	01B, 02B, 03B	1	4	X	X	X
B	2	04B, 05B, 06B	2	5	X		X
B	3	07B, 08B, 09B	1	6	X	X	

Three orthopox positive NHP blood samples (obtained from days 2, 5, and 8 post *Monkeypox virus* exposure) obtained from a previous animal study (Huggins, unpublished) were chosen to represent low, medium and high viral genomic titers, respectively. DNA was extracted previously and GC values were determined while the animal study was in progress. Standards, controls, and test samples were assayed on eight of nine runs. Volumes for D2 (low), D5 (medium) and D8 (high) test samples were not of sufficient volume to complete the ninth run (09) of the Set A assays. The ninth run was completed with a substitute sample, but the data was not utilized for determination of precision. Instead, the standard curve data was collected and applied to the Verification of the Standard Curve data set. Since nine runs were required to evaluate intermediate precision, high, medium and low test samples for Set B assays were prepared by spiking negative control serum (NCS) with stock *Monkeypox virus* (1 × 10^8^, 1 × 10^6^ and 1 × 10^4^ pfu), extracted, and run on the LightCycler.

This assay showed acceptable levels of precision for all standards and test samples with ≥ 50 GC/5uL (10,000 GC/mL). All mean GC values for each acceptable HA standard as determined for each assay had a %CV ranging from 0.52 to 21.88, passing the acceptance criteria for repeatability and intermediate precision (Additional file 1: Tables S1 and S2). Positive test samples that were above the lower limit of quantitation (LLOQ, see “Accuracy”) that ranged from 4.93 × 10^3^ to 7.42 × 10^6^ GC/5uL also exhibited acceptable repeatability (with the exception of assay #1) and intermediate precision. In terms of repeatability, the %CV for all PTS with medium range GC values ranged from 8.82 to 35.40. The %CV for all PTS with high range GC values ranged from 10.41 to 31.97 (Additional file 1: Table S1). For intermediate precision testing, %CV for PTS with medium range and high GC values ranged from 15.13 to 28.90% and 15.67 to 25.40%, respectively (Additional file 1: Table S2).

All positive extraction values tested in duplicate for each run met the acceptance criteria as established in Table 1. The %CV for all PEC tested in the 6 repeatability assays ranged from 22.30 to 42.15 (Additional file 1: Table S1). The %CV of the PEC in all four intermediate precision assays ranged from 22.20 to 48.30. The PEC failed repeatability and intermediate precision (same analyst) in Assay #3 (%CV of 42.15%, Additional file 1: Table S1) and Assay 1/3 (%CV of 48.30%, Additional file 1: Table S2), respectively. Since the PEC is primarily used to verify that samples are extracted properly, we deemed it more important that the values for the PEC fall within the predetermined range (1.58 × 10^3^ to 1.58 × 10^4^ GC/5uL). Furthermore, the high %CV appeared to be due to technician error. Together, the overall precision of this assay utilizing the HA standards, PTS, and PEC is acceptable for its intended use.

### Verification of selectivity

Selectivity, as defined by FDA guidance, is “…the ability of an analytical method to differentiate and quantify the analyte in the presence of other components in the sample” [6]. In this study, the other components are those remaining in the extracted blood. Therefore, selectivity was verified by spiking 14 negative test samples (NTS1–14) with *Monkeypox virus* DNA (NCS spiked with 1.1 × 10^5^ GC/5 μl). Negative test samples (NTS) were first assayed to verify the presence or absence of orthopox DNA and dropped if orthopox contamination (>LLOQ) was present. The GC value of the spiked water sample provided the reference value used for calculating recovery. Three assays, each consisting of 2 runs were completed. Given the intended use of the assay the selectivity was deemed acceptable when the recovery of each spiked sample was between 80 and 120%.

After spiking with viral DNA (approximately 1 × 10^5^ GC), 4 of 12 tested samples gave rise to recovery values (75%, 75%, 77%, 75%) below the limits set in the acceptance criteria (Additional file 1: Table S3). The remaining spiked samples resulted in recovery values ranging from 80% to 87%. Based on this observation, matrix effects likely had a dampening effect on all samples tested. Based on the data (Additional file 1: Table S3), one could reasonably expect, and should account for, at least a 13% loss in signal and as much as a 25% loss.

### Verification of accuracy

“The accuracy of an analytical method describes the closeness of mean test results obtained by the method to the true value (concentration) of the analyte.” as defined by the FDA [6]. Titration of poxvirus(es) by plaque assay is traditionally implemented to determine viral load (the amount of virus in blood or tissue). This method depends strongly on the quality and consistency of a live culture system (cell culture), requires multiple days before data can be acquired, and has limited sensitivity. In the case of *Monkeypox virus*, material must be handled in a biological safety level three (BSL-3) laboratory by properly trained individuals, whereas inactivating the samples allows subsequent processing in a more accessible environment. Inactivating the sample has drawbacks, namely, determination of infectious units is no longer possible and the integrity of nucleic acids may be affected.

In our study three methods, identified as A, B, and C, were used to test accuracy (Table 3). Each method consisted of 3 assays and each assay consisted of 3 Lightcycler runs with an acceptable recovery range of 50–150%. This range was purposefully broad to represent the application for which it was intended, that is, quantitation of viremia from monkeypox exposed cynomolgus macaques. It is unlikely that a potential therapeutic will be judged as efficacious by a slim margin in viremia compared to placebo or another treatment, or, for that matter, by viremia alone. Therefore we found this range to be more in line with a “real world scenario” of meaningful viral DNA quantity comparisons.

**Table 3:** An overview of accuracy testing by three methods

Set	Method	Assay (3 LC runs per assay)	Reference Values
A	Negative blood spiked with MPX virus	1, 2, 3	Converted pfu to GC/ml
B	Negative blood spiked with extracted DNA	1, 2, 3	Used GC values from Set A
C	Negative blood spiked with extracted DNA	1, 2, 3	Used GC values from Set C

Method A used NCS spiked with a 1 to 10 serial dilution (1 × 10^3^ pfu/ml to 1 × 10^8^ pfu/ml) of stock *Monkeypox virus*. The spiked NCS was extracted and amplified on the LightCycler (9 runs). Mean GC values were determined for each assay and compared to the reference values. The reference value, as determined by plaque assay titration (data not shown) was converted from pfu/mL to GC/5 μl by dividing the pfu/ml of the diluted virus stock samples by 200 and then multiplying by a previously resolved conversion factor(13) which was determined as follows: *Monkeypox virus* was spiked, and serially diluted, into cynomolgus blood for a total of 9 dilution series performed over 3 days. Both quantitative PCR assay (subsequent to extraction) and plaque titrations were performed. For each dilution, a genome to pfu ratio was calculated. A mean of the resulting genome to pfu ratios was calculated(data not shown). Based off this calculated reference value, the mean recovery value per assay was determined for each sample. Of the concentrations evaluated (NCS spike with 10^8^ pfu/mL to 10^3^ pfu/mL), all but 1/3 NCS spiked with 10^4^ pfu/mL (2/3 assays, Additional file 1: Table S4) passed recovery. This was most likely due to technical error as samples at lower spiked concentrations met criteria and were acceptable.

Method B avoided the conversion of pfu to GC. Extracted DNA from method A was prepared for spiking NCS to give an approximate range of 1 × 10^3^ to 1 × 10^8^ GC/ml. The spiked NCS was extracted and tested in 9 runs. The GC values for reference standards used in Set B were calculated from serial dilutions of extracted DNA obtained in Set A. The mean recovery value per sample was determined for each assay. For this method, NCS spiked at 1 × 10^5^ GC/ml through 1 × 10^8^ GC/ml met all of the acceptance criteria for each run and passed all recovery tests. The 1 × 10^4^ GC/ml and 1 × 10^3^ GC/ml material failed recovery in 1 of 3 and 2 of 3 assays, respectively (Additional file 1: Table S4).

Because there was a lack of consistency in the 1 × 10^3^ GC/ml spiked samples, we repeated the method with a single modification. Method C also used NCS spiked with extracted DNA from method A, but the reference material was extensively tested to yield a more exact concentration. After dilution, the reference material that was consistent with the acceptance criteria was deemed suitable as Reference samples. Dilutions that did not fall within acceptable ranges were dropped from further use. NCS was spiked with the Reference samples, extracted and tested in 9 runs. The mean recovery value per assay was determined for each sample. Here, the NCS spiked with 9.92 × 10^0^, 9.92 × 10^2^, 9.92 × 10^3^, 8.41 × 10^4^, and 8.41 × 10^5^ GC met all of the acceptance criteria for each run. All spiked NCS passed recovery testing in all assays with values ranging from 50% (49.76%) to 117% (116.51%) (Additional file 1: Table S4).

It should be noted that for samples < 50 GC, the %CV per run was often above our threshold (≤ 30%). This is not surprising as this is below our demonstrated limit of quantification (as will be discussed in the next section).

### Verification of the standard curve

A standard curve with defined detection and quantifiable limits (i.e., ULOQ, LLOQ, and LOD) was a requirement for the Orthopox assay validation. The cloned *Variola virus* strain Bangladesh (VARV-BSH) HA gene was used as the standard curve for this assay. The sequences used within the HA gene are well conserved among orthopoxviruses [4]. Although the preference was to spike *Monkeypox virus* into nonhuman primate blood, we chose to use the HA standard after considering the potential biological safety and logistical issues with this procedure. Furthermore, the DNA standard could be produced in bulk and concentrations established by established, accepted methods (e.g., spectrophotometry) ensuring less variation between lots.

Three assays, each comprised of 3 LightCycler runs, were performed to test consistency and identify the limits of detection (LOD) and quantitation (LOQ). The upper and lower limit of quantitation (ULOQ and LLOQ) is the highest and lowest amount of an analyte in a sample that can be quantified with acceptable precision and accuracy, respectively (8). Concentrations of HA ranging from 1 × 10^−1^GC/5ul to 1 × 10^10^ GC/5 μl (samples bsh-ha-0.1 to bsh-ha-9.0) were prepared and evaluated in triplicate (Additional file 1: Table S5). Recovery values and CVs were determined for each concentration. In order to meet the criteria, the LLOQ and ULOQ must be detectable in 3 out of 3 tests (9 runs), have a %CV ≤ 30, and recovery between 80% and 120%. The stringent recovery range was implemented due to the fact that the HA standard was not being extracted. The LOD was defined as the number of genomes resulting in detection in 2 out of 3 tests (6 out of 9 runs). Stability of the HA Standards over time was determined from the mean daily efficiency values for the entire study (Additional file 1: Table S6).

The HA Standard containing approximately 2.5 GC/5 μl was detected in 6 out of 9 runs and met the criteria for the LOD (Additional file 1: Table S5). The HA Standard dilution containing approximately 5 × 10^1^ GC met the LLOQ criteria by detection in 3 out of 3 tests (9 of 9 runs), a %CV of 22.23, 13.40, and 12.94; and a mean recovery value of 106. The HA Standard dilution containing approximately 5 × 10^7^ GC met the ULOQ criteria by detection in 3 out of 3 tests, a %CV of 1.81, 2.44, and 3.03; and a mean Recovery value of 117. The mean daily efficiency values of the HA Standards ranged from 1.911 to 1.998 with a %CV ranging from 0.050 to 3.433 (Additional file 1: Table S6). In terms of stability of the standard curve, the mean efficiency value for the entire study was 2.0 with a %CV of 1.1, suggesting stability of the HA standards for at least 92 days.

### Verification of stability

Stability testing examines the precision of the assay (and components) over time or when variations are introduced. The primary concerns for this assay were the potential effect of freeze/thaw cycles on the HA DNA standard and the long term stability of the PEC and PCR Master Mix (MGB Master Mix).

Three assays were used to test the stability of the *pan*-orthopox virus HA MGB assay. The first assay tested viral DNA stability by evaluating the freeze thaw effect on GC values. Three concentrations of HA standards (5 × 10^2^, 5 × 10^4^ and 5 × 10^6^ GC/5 μL), PEC, and PTS were freeze thawed 10 times and tested following the 1st, 5th, and 10th freeze thaws. The PEC was extracted prior to freeze thaw and the PTS was extracted following freeze thaw. The %CV was determined from the 3 runs for each sample. The second assay tested PEC stability over time by comparing GC values from a previous protocol to that of a current lot, which was prepared 4 months later. Two samples from each lot were tested in duplicate per run and the CV was determined for the mean GC values. The third assay tested the stability of MGB Master Mix by comparing the GC values of HA Standards (5 × 10^2^, 5 × 10^4^ and 5 × 10^6^ GC/5uL) assayed with two different lots of Master Mix that were temporally separated by 8 months. The CV was determined from the 3 HA Standards tested in duplicate.

All of the assays met acceptance criteria. The 3 concentrations of HA Standards subjected to freeze-thaw had %CVs of 4.52, 1.94, and 11.75 (Additional file 1: Table S7). The PEC and PTS subjected to freeze-thaw had %CVs of 35.75 and 20.86, respectively. The %CV from testing different lots of PEC was 17.01. Preparing 3 concentrations of HA Standards with different lots of MGB Master Mix resulted in %CVs of 12.87, 11.58, and 16.61. Together, these data support that there is little or no effect of freeze thaw cycles on the HA standard and the PEC and MGB Master mix(es) are stable for at least 4 and 8 months, respectively.

### Verification of specificity

Specificity tests the ability of the assay to identify a specific analyte within the test matrix. In this section, orthopox virus DNA was the analyte and MGB Master Mix was the matrix. DNA isolated from *Herpes simplex virus*-1 (HSV-1), *Herpes simplex virus-2* (HSV-2), *Camelpox*, *Vaccinia*, *Rabbitpox*, and *Cowpox viruses* were obtained from the Diagnostic Systems Division, at USAMRIID. A more extensive panel has been tested [4], therefore we focused on DNA samples that were representative of viruses being utilize in our lab. A 1:100 dilution of each isolate was prepared and both the diluted and undiluted preparations were tested in duplicate using the MGB Master Mix.

All MGB Master Mix samples spiked with orthopox virus DNA (*Camelpox*, *Vaccinia*, *Rabbitpox*, and *Cowpox virus* DNA), tested positive (Additional file 1: Table S8). MGB Master Mix samples spiked with non-orthopox viral DNA, HSV-1 and the diluted sample of HSV-2, tested negative. The undiluted sample of HSV-2 gave rise to low levels (3 to 8 GC/5 ul) of Orthopox GC. Further testing utilizing a monkeypox specific PCR assay [7] indicated that the HSV-2 sample contained monkeypox DNA contamination (data not shown).

### Verification of robustness

Robustness tests the ability of an assay to remain unaffected by small but deliberate changes and provides an indication of its reliability during normal usage. Three assays, each consisting of 2 LightCycler runs, were used to test the effect of 2 lots of MGB Master Mix, 2 lots of Qiagen extraction kits, and 2 LightCyclers, on the GC values (Table 4). HA Standards and aliquots of the PEC were used to test 2 lots of MGB Master Mix by determining the %CV of GC values from LightCycler runs VP42 and VP44 (Lot B) and again from runs VP43 and VP45 (Lot A), where the other parameters (extraction kit lot and Lightcycler instrument) were held constant. We found that all of the VARV-BSH standards and the three aliquots of PEC met the acceptance criteria (Table 1) with a %CV ranging from 1.32 to 23.39 and 23.60 to 39.86, respectively (Additional file 1: Table S9). The PEC failed the acceptance criteria in assay #1 with a %CV of 47.44 but passed in assay #2 with a %CV of 22.78.

**Table 4:** Overview of robustness testing

Assay ID	Extraction Kits	MGB Master Mix-orthopox	LightCycler Instruments Serial #
VP42	Lot#1	Lot B	1403531
VP42B	Lot#1	Lot B	1403531
VP43	Lot#1	Lot A	1403531
VP43B	Lot#2	Lot B	1403531
VP44	Lot#1	Lot B	1403649
VP45	Lot#1	Lot A	1403649

Aliquots of the PEC were used to test different lots of Qiagen kits by determining the %CV of GC values from runs VP42B and VP43B in which only the Qiagen kit lot was changed. Each aliquot of the PEC was prepared by extracting over a column from one of the two kits. Aliquots A, B, and C were extracted using “Lot #1” whereas D, E, and F were extracted with “Lot #2”. Aliquots A, B, and C were tested in duplicate in run VP42B and aliquots D, E, and F were tested in duplicate in run VP43B. Aliquot B had a GC value of 4.07 × 10^4^ whereas the other aliquots had values ranging from 1.20 × 10^4^ to 1.67 × 10^4^. Aliquot B was dropped from further calculations as an outlier using Dixon’s Gap Analysis [8]. The %CV of the mean GC value for all aliquots in each assay was 18% and 8.7%, and for both assays was 13%, meeting the acceptance criteria (Table 1 and Additional file 1: Table S10).

HA Standards and aliquots of the PEC were used to test the two LightCyclers by determining the %CV of GC values from runs VP42 and VP44 and again from runs VP43 and VP45 (Table 4). All tests met the acceptance criteria with %CV ranging from 1.06 to 25.6% (Additional file 1: Table S11).

From the robustness testing, we found that implementing different Master Mix lots, Qiagen extraction kit lots, or changing instruments did not impact the performance of the assay.

## Discussion

There are no FDA approved therapeutics with a specific indication for the treatment of smallpox or monkeypox disease. Two orally available investigational compounds, tecovirimat and brincidofovir, were granted fast-track status by the FDA and could potentially fulfill a mandate for smallpox therapeutics to be included in the Strategic National Stockpile (reviewed by [9]). While both compounds have been evaluated for safety and tolerability in human clinical studies, the lack of human smallpox cases and severely austere conditions in areas where monkeypox is endemic make human efficacy trials supporting a smallpox indication extremely unlikely.

Under U.S. regulations, when human efficacy studies are not ethical or feasible the effectiveness of drug or biological products may be evaluated in an appropriate animal model or models. However, as is the case with traditional product approvals, it is critical to ensure the validity of data and appropriateness of assays used to support regulatory decision making. For example, assay performance is called out several times in the 2015 FDA Animal Rule guidance: assays used to quantify the challenge agent dose should be validated, and those used to detect biomarkers used as a trigger-to-treat or as the basis for human dose selection should be adequately described. The methodology described within this manuscript has supported the development of tecovirimat and brincidovovir within the confines of the validation for the intravenous monkeypox model [1, 2]. The presence of circulating virus immediately post-exposure is used as an indicator of successful exposure, with the actual concentration of virus used as an acceptance criteria in the model [9]. Virema is also monitored during disease progression, and can be useful for linking clinical improvement to a decrease in circulating virus levels following treatment with antivirals. A well-characterized assay allows comparisons to be made not only between study groups but also across studies to demonstrate robustness of the intravenous challenge model.

Acceptance criteria were not met for accuracy, selectivity, and CV at low GC values. In most cases we rectified the problem through additional, more refined study (e.g., accuracy), in other cases (e.g., selectivity and CV at low GC values), this was not possible because we overestimated the ability of the method. In retrospect, more data collection prior to the validation study most likely would have eliminated these issues, but the validation was deemed acceptable for its intended use given the limitations of the assay at low GC values and the high levels of viremia observed in the IV monkeypox model. A summary of the validation can be found in Table 5.

**Table 5:** Summary of findings for Orthopox DNA PCR

Parameter	Conclusion
Precision	Acceptable when GC/5 μL is ≥ LLOQ
Repeatability	
Intermediate	
Selectivity	Matrix effects of 13–25%
Accuracy	within 50–150% (when ≥ LLOQ)
Standard Curve	LOD: 2.5 GC/5 μL
	LLOQ: 50 GC/5 μL
	ULOQ: 5 × 10^7^ GC/5 μL
	Efficiency: 2.0 ± 0.2
	Stability: ≥ 92 days
Stability	
HA standards	stable after 10 freeze thaws
PEC	stable for at least 4 months
Master Mix	stable for at least 8 months
Specificity	orthopox specific; see also [4]
Robustness	no/little effect when changing extraction kit lots, Master Mix lots, or LightCycler instruments

Depending on the requirements of the end-user, certain areas of the method performance could be refined. Improvement could focus on further optimization of the extraction process. Further optimization (increasing efficiency and /or decreasing contaminants) would likely provide the most benefit as it impacts the downstream PCR process. Improved extraction would greatly increase selectivity and possibly narrow the somewhat large acceptance criteria for accuracy and/or improve the performance of the assay at low levels of viral nucleic acid.

Our method performed particularly well in areas such as stability, specificity, and robustness, and was accurate at levels of viremia likely to be of clinical significance. DNA was stable after multiple freeze thaws and both the positive extraction control and mastermix were stable for 4 and 8 months, respectively. The selectivity of the method is of interest as viremia can be directly attributed to *Monkeypox virus* (and not any potential laboratory contaminants), a feature that direct viral titration does not offer.

The ability to quantitate and compare the magnitude of circulating viral genome with a stringent and controlled method allows for a direct comparison of viremia levels in alternative models, as well as in the intravenous monkeypox model itself. This assay has been used to demonstrate the in vivo fitness of recombinant poxviruses and for poxvirus models using alternative exposure routes [10, 11]. This assay has also been utilized to support countermeasure development in the intravenous variola model [2, 9]).

The method we describe here has been validated for its primary purpose, that is, to support medical countermeasure development in the intravenous monkeypox model. We have applied our method to other nonhuman primate poxvirus infection model systems (e.g., marmosets and rhesus macaques), but further studies would be required to validate the assay for these alternative models [12, 13]. If field trials require assaying human blood for MPXV in endemic areas, a nucleic acid based assay would be highly desirable given that classical virus titration in mammalian cell culture under those austere conditions would be extremely challenging.

## Conclusion

Here we present a quantitative real time PCR assay validated for the determination of monkeypox viral GC levels in cynomolgus macaque EDTA whole blood (Table 5). The assay was designed to support the near-real-time monitoring of viral load and to facilitate comparison of viral load in nonhuman primate studies. Since validation is an essential component of a GLP study environment, it was necessary to establish the operational parameters of this assay. Precision, selectivity, accuracy, limits of detection, stability, specificity, and robustness were examined utilizing samples representative of those obtained during normal experimental procedures. HA Standards and test samples demonstrated acceptable levels of precision (≤ 30% CV**)** with ≥ 50 GC/5 μL of blood. The detection of PTS with GC values ≤ 50 and high CVs noted throughout the study further supported the standard curve data for placing the LLOQ at 50 GC/5 μl and the LOD at 2.5 GC/5 μl. PEC values with %CV > 40% were deemed acceptable due to this control serving as verification for the extraction procedure. Matrix effects due to the components that remained from extracted cynomologus macaque blood were detected, suggesting the selectivity of the assay can be influenced by substances that are co-extracted with the DNA and that GC values are actually higher (≤ 25%) than measured. High PCR sensitivity resulted in technical problems for the first two methods attempted to test the accuracy but the third method clearly shows GC ≥ 10 /5 μl meeting the acceptance criteria. Since a wide range of virema occurs during poxvirus infection (from 0 GC/ml in uninfected controls to > 10^7^ GC/ml in animals with severe disease), a recovery of 50 to 150% is sufficient for detecting differences ≥0.5 log. The amplification was specific for orthopox virus genomes, and identified a previously unsuspected poxvirus contamination of a HSV-2 sample. Based on the validation results, the *pan*-orthopox HA MGB assay can be used to assess orthopox viral load by quantitating the copies of HA gene present in a NHP (cynomolgus macaque) EDTA blood sample. Given the need for regulated studies and supporting validated assays for the evaluation of orthopox countermeasures, our *pan*-orthopox HA MGB assay (in the context of this validation) will be integral for the progression and potential FDA licensing of current and future orthopox therapeutics.

## Additional files

Additional file 1:Table S1. Repeatability for each of the 6 assays. *N* = 3 unless stated otherwise. Table S2. Intermediate precision testing. *N* = 6 unless stated otherwise. Table S3. Selectivity testing of spiked NTS. Table S4. Accuracy testing by 3 methods. (*N* = 3 per assay). Table S5. Standard curve assessment. Table S6. Stability of the HA Standard. Table S7. Stability testing of HA Standard, PEC, and MGB Master Mix. Table S8. Specificity testing using different viral DNA. Table S9. Ruggedness testing of MGB Master Mix. Table S10. Ruggedness testing of extraction kits. Table S11. Ruggedness testing using two LightCyclers instruments. (DOCX 53 kb)

## Abbreviations

BSH: Bangladesh
CDC: Center for Disease Control and Prevention
CFR: Code of Federal Regulations
CT: Crossing threshold/crossing point
CV: Coefficient of variation
D: Day
DNA: Deoxyribonucleic acid
FDA: U.S. Food and Drug Administration
GC: Genome copies
GLP: Good Laboratory Practices
HA: Hemagglutinin
HSV: *Herpes simplex virus*
IN: Indiana
LLOQ: Lower limit of quantitation
LOD: Limit of detection
MGB: Minor groove binding
mL: Milliliter
MPX: Monkeypox
NC: Negative control
NCS: Negative control serum
NHP: Nonhuman primate
PCR: Polymerase chain reaction
PEC: Positive extraction control
PTS: Positive test sample
QARCO: Quality Assurance and Regulatory Compliance Office
SD: Standard deviation
U.S.: United States
ULOQ: Upper limit of quantitation
USAMRIID: United States Army Medical Research Institute of Infectious Diseases
VarV: *Variola virus*
VECTOR: State Research Center and Biotechnology, Novosibirsk, Russia
μL: Microliter
