# Supplementary material for: Validation of a pan-orthopox real-time PCR assay for the detection and quantification of viral genomes from nonhuman primate blood
Source: Virol J. 2017 Nov 3;14:210. doi: 10.1186/s12985-017-0880-8 (PMC5670720; doi:10.1186/s12985-017-0880-8)
Supplement: Supplementary file 1 — Repeatability for each of the 6 assays. N = 3 unless stated otherwise. Table S2. Intermediate precision testing. N = 6 unless stated otherwise. Table S3. Selectivity testing of spiked NTS. Table S4. Accuracy testing by 3 methods. (N = 3 per assay). Table S5. Standard curve assessment. Table S6. Stability of the HA Standard. Table S7. Stability testing of HA Standard, PEC, and MGB Master Mix. Table S8. Specificity testing using different viral DNA. Table S9. Ruggedness testing of MGB Master Mix. Table S10. Ruggedness testing of extraction kits. Table S11. Ruggedness testing using two LightCyclers instruments. (DOCX 53 kb) [file 12985_2017_880_MOESM1_ESM.docx]

**Supplemental Data**

**Supplemental Table 1:** **Repeatability for each of the 6 assays. N = 3 unless stated otherwise**.

|  | Assay 1 | | Assay 2 | | Assay 3 | | Assay 1B | | Assay 2B | | Assay 3B | |
| --- | --- | --- | --- | --- | --- | --- | --- | --- | --- | --- | --- | --- |
|  | Mean GC/5uL | % CV | Mean GC/5uL | % CV | Mean GC/5uL | % CV | Mean GC/5uL | % CV | Mean GC/5uL | % CV | Mean GC/5uL | % CV |
| Sample |  |  |  |  |  |  |  |  |  |  |  |  |
| 5x 10^6^ HA Standard | 4.91x 10^6^ | 3.37 | 4.97x 10^6^ | 1.5 | 5.03x 10^6^ | 1.04 | 5.31x 10^6^ | 2.76 | 5.23x 10^6^ | 1.1 | 5.26x 10^6^ | 0.7 |
| 5x 10^5^ HA Standard | 4.99x 10^5^ | 1.91 | 5.01x 10^5^ | 3.85 | 4.97x 10^5^ | 3.85 | 4.54x 10^5^ | 1.79 | 4.56x 10^5^ | 2.48 | 4.49x 10^5^ | 1.73 |
| 5x 10^4^ HA Standard | 5.31x 10^4^ | 6.01 | 5.11x 10^4^ | 2.12 | 5.06x 10^4^ | 4.31 | 5.25x 10^4^ | 1.14 | 5.26x 10^4^ | 1.32 | 5.30x 10^4^ | 0.99 |
| 5x 10^3^ HA Standard | 4.55x 10^3^ | 7.75 | 5.10x 10^3^ | 6.05 | 4.89x 10^3^ | 1.01 | 4.48x 10^3^ | 5.55 | 4.37x 10^3^ | 5.32 | 4.48x 10^3^ | 1.01 |
| 5x 10^2^ HA Standard | 4.84x 10^2^ | *5.12 | 5.23x 10^2^ | 1.65 | 5.10x 10^2^ | 1.19 | 5.58x 10^2^ | 3.27 | 5.71x 10^2^ | 2.56 | 5.85x 10^2^ | 1.2 |
| 5x 10^1^ HA Standard | 5.47x 10^1^ | 21.88 | 4.91x 10^1^ | 1.26 | 4.91x 10^1^ | 2.8 | 4.92x 10^1^ | 2.26 | 4.82x 10^1^ | 0.81* | 4.74x 10^1^ | 0.52* |
| PEC | 4.63x 10^3^ | 39.57 | 4.11x 10^3^ | 22.99 | 2.60x 10^3^ | **42.15** | 6.79x 10^3^ | 24.84 | 7.31x 10^3^ | 23.98 | 8.15x 10^3^ | 22.3 |
|  |  |  |  |  |  |  |  |  |  |  |  |  |
| D8 (high) | 1.79x 10^5^ | **31.97** | 1.68x 10^5^ | 17.17 | 1.57x 10^5^ | 11.75* | NA | NA | NA | NA | NA | NA |
| D5 (medium) | 5.86x 10^3^ | **35.4** | 5.48x 10^3^ | 19.2 | 4.93x 10^3^ | 13.07* | NA | NA | NA | NA | NA | NA |
| D2 (low) | 2.02x 10^0^ | **34.08** | 1.58x 10^0^ | **64.93** | 2.87x 10^0^ | **53.39*** | NA | NA | NA | NA | NA | NA |
| NCS + virus (high) | NA | NA | NA | NA | NA | NA | 7.42x 10^6^ | 10.41 | 6.28x 10^6^ | 18.21 | 6.84x 10^6^ | 24.84 |
| NCS + virus (medium) | NA | NA | NA | NA | NA | NA | 6.29x 10^4^ | 8.82 | 5.65x 10^4^ | 21.12 | 6.42x 10^4^ | 23 |
| NCS + virus (low) | NA | NA | NA | NA | NA | NA | 2.97x 10^1^ | 23.66 | 2.47x 10^1^ | **83.81** | 3.05x 10^1^ | **49.03** |

Bold font indicates results outside range of acceptance criteria.

* Indicates N=2

**Supplemental Table 2:** **Intermediate precision testing. N = 6 unless stated otherwise**.

|  |  | Same Analyst | | | |  | Different Analyst | | | |
| --- | --- | --- | --- | --- | --- | --- | --- | --- | --- | --- |
|  |  | Assay 1 & 3 | | Assay 1B & 3B | |  | Assay 1 & 2 | | Assay 1B & 2B | |
| Sample |  | Mean GC/5 μl | %CV | Mean GC/5 μl | %CV |  | Mean GC/5 μl | %CV | Mean GC/5 μl | %CV |
|  |  |  |  |  |  |  |  |  |  |  |
| Standard 5x 10^6^ |  | 4.97x 10^6^ | 2.57 | 5.28x 10^6^ | 1.88 |  | 4.94x 10^6^ | 2.43 | 5.27x 10^6^ | 2.07 |
| Standard 5x 10^5^ |  | 4.98x 10^5^ | 2.73 | 4.51x 10^5^ | 1.67 |  | 5.00x 10^5^ | 2.73 | 4.55x 10^5^ | 1.96 |
| Standard 5x 10^4^ |  | 5.19x 10^4^ | 5.38 | 5.27x 10^4^ | 1.08 |  | 5.21x 10^4^ | 4.59 | 5.25x 10^4^ | 1.11 |
|  |  |  |  |  |  |  |  |  |  |  |
| Standard 5x 10^3^ |  | 4.72x 10^3^ | 6.2 | 4.48x 10^3^ | 3.56 |  | 4.83x 10^3^ | 6.06 | 4.43x 10^3^ | 5.06 |
|  |  |  |  |  |  |  |  |  |  |  |
| Standard 5x 10^2^ |  | 5.01x 10^2^ | 3.55 | 5.71x 10^3^ | 3.44 |  | 5.07x 10^2^ | 4.51 | 5.64x 10^2^ | 2.91 |
|  |  |  |  |  |  |  |  |  |  |  |
| Standard 5x 10^1^ |  | 4.86x 10^1^ | 15.71 | 4.85x 10^2^ | 2.56 |  | 2.85x 10^1^ | 2.66 | 4.88x 10^1^ | 2 |
|  |  |  |  |  |  |  |  |  |  |  |
| PEC |  | 3.61x 10^3^ | **48.3** | 7.47x 10^3^ | 23.26 |  | 4.37x 10^3^ | 30.52 | 7.05x 10^3^ | 22.2 |
|  |  |  |  |  |  |  |  |  |  |  |
| D8 (high) |  | 1.70x 10^5^ | 25.40* | NA | NA |  | 1.73x 10^5^ | 23.62 | NA | NA |
|  |  |  |  |  |  |  |  |  |  |  |
| D5 (medium) |  | 5.48x 10^3^ | 28.90* | NA | NA |  | 5.67x 10^3^ | 26.2 | NA | NA |
|  |  |  |  |  |  |  |  |  |  |  |
| D2 (low) |  | 2.36x 10^0^ | **43.14*** | NA | NA |  | 1.80x 10^0^ | **45.44** | NA | NA |
|  |  |  |  |  |  |  |  |  |  |  |
| NCS + virus (high) |  | NA | NA | 7.13x 10^6^ | 17.14 |  | NA | NA | 6.85x 10^6^ | 15.67 |
|  |  |  |  |  |  |  |  |  |  |  |
| NCS + virus (medium ) |  | NA | NA | 6.35x 10^4^ | 15.73 |  | NA | NA | 5.97x 10^4^ | 15.13 |
|  |  |  |  |  |  |  |  |  |  |  |
| NCS + virus (low ) |  | **NA** | NA | 3.01x 10^1^ | **34.71** |  | NA | NA | 2.72x 10^1^ | **51.81** |

Bold font indicated results outside range of acceptance criteria.

* indicates N=5.

**Supplemental Table 3:** **Selectivity testing of spiked NTS**

| **Test Sample** | **N** | **% Recovery** |
| --- | --- | --- |
| NTS1 + PTS | 4 | 83 |
| NTS2 + PTS | 4 | **75** |
| NTS3 + PTS | 4 | **75** |
| NTS4 + PTS | 4 | 83 |
| NTS5 + PTS | 4 | 87 |
| NTS6 + PTS | 4 | 80 |
| NTS7 + PTS | 4 | 82 |
| NTS8 + PTS | 4 | 80 |
| NTS11 + PTS | 4 | **75** |
| NTS12 + PTS | 4 | 84 |
| NTS13 + PTS | 4 | 84 |
| NTS14 + PTS | 4 | **77** |

Bold font indicated results outside range of acceptance criteria.

**Supplemental Table 4:** **Accuracy testing by 3 methods. (N=3 per assay)**

| Reference Samples | | Assay: #1  % Recovery | #2  %  Recovery | #3  %  Recovery | Spiked  NCS  N=9 |
| --- | --- | --- | --- | --- | --- |
| Method A | |  |  |  |  |
| ~pfu/ml | ~GC/5μl |  |  |  | GC/5μl |
| 1x 10^8^ | 6.5x 10^6^ | 56.90 | 67.34 | 90.05 | 4.65x 10^6^ |
| 1x 10^7^ | 6.5x 10^5^ | 78.67 | 67.64 | 98.10 | 5.30x 10^5^ |
| 1x 10^6^ | 6.5x 10^4^ | 97.23 | 81.23 | 80.21 | 5.60x 10^4^ |
| 1x 10^5^ | 6.5x 10^3^ | 78.87 | 64.01 | 107.87 | 5.43x 10^3^ |
| 1x 10^4^ | 6.5x 10^2^ | **41.67** | **44.64** | 50.18 | 2.96x 10^2^ |
| 1x 10^3^ | 6.5x 10^1^ | 53.23 | 92.54 | 94.38* | 5.20x 10^1^ |
|  |  |  |  |  |  |
| Method B | |  |  |  |  |
| ~GC/ml | ~GC/5μl |  |  |  |  |
| 1x 10^8^ | 5.0x 10^5^ | 50.53 | 85.87 | 97.46 | 4.02x 10^5^ |
| 1x 10^7^ | 5.0x 10^4^ | 50.73 | 74.73 | 102.16 | 3.76x 10^4^ |
| 1x 10^6^ | 5.0x 10^3^ | 61.37 | 105.73 | 94.96 | 4.90x 10^3^ |
| 1x 10^5^ | 5.0x 10^2^ | 58.33 | 95.37 | 76.72 | 4.58x 10^2^ |
| 1x 10^4^ | 5.0x 10^1^ | **39.57** | 80.00 | 89.01 | 1.05x 10^2^ |
| 1x 10^3^ | 5.0x 10^0^ | **13.97** | 74.32 | **339.57** | 2.04x 10^1^ |
| Method C  GC/5μl | |  |  |  |  |
| 8.41x 10^5^ | | 81.59 | 92.89 | 97.46 | 7.62x 10^5^ |
| 8.41x 10^4^ | | 85.14 | 108.52 | 102.16 | 8.29x 10^4^ |
| 9.92x 10^3^ | | 78.99 | 91.09 | 94.96 | 8.77x 10^3^ |
| 9.92x 10^2^ | | 52.45 | 64.10 | 76.72 | 6.40x 10^2^ |
| 9.92x 10^1^ | | 49.76 | 70.31 | 89.01 | 6.91x 10^1^ |
| 9.92x 10^0^ | | 70.58 | 101.33 | 116.51 | 5.98x 10^0^ |

Bold font indicated results outside range of acceptance criteria.

*N=2,

**Supplemental Table 5:** **Standard curve assessment**

| Conc. of | Assay #1 | | | Assay #2 | | | | Assay #3 | | | | | MEAN |
| --- | --- | --- | --- | --- | --- | --- | --- | --- | --- | --- | --- | --- | --- |
| Test Samples  GC/5μl | Det | Mean  GC/5μl | %  CV | Det | Mean  GC/5μl | %  CV | | Det | | Mean  GC/5μl | %  CV | | %  Recovery |
| Bsh-ha-9.0 | 0/3 | Out of range | NA | 0/3 | Out of range | NA | | 0/3 | | Out of range | NA | | NA |
| Bsh-ha-8.0 | 3/3 | 6.64x 10^8^ | 0.57 | 3/3 | 5.76x 10^8^ | 0.30 | 3/3 | | 5.77x 10^8^ | | 2.76 | **121.18** | |
| Bsh-ha-7.0 | 3/3 | 6.61x 10^7^ | 1.81 | 3/3 | 5.65x 10^7^ | 2.44 | | 3/3 | | 5.36x 10^7^ | 3.03 | | 117.49 |
| Bsh-ha-6.0 | 3/3 | 6.20x 10^6^ | 0.97 | 3/3 | 5.17x 10^6^ | 1.02 | | 3/3 | | 5.44x 10^6^ | 2.30 | | 112.07 |
| Bsh-ha-5.0 | 3/3 | 5.30x 10^6^ | 0.61 | 3/3 | 5.47x 10^5^ | 1.87 | | 3/3 | | 5.50x 10^5^ | 3.15 | | 108.51 |
| Bsh-ha-4.0 | 3/3 | 5.37x 10^5^ | 0.65 | 3/3 | 5.48x 10^4^ | 1.11 | | 3/3 | | 5.66x 10^4^ | 1.41 | | 110.04 |
| Bsh-ha-3.0 | 3/3 | 5.70x 10^4^ | 1.47 | 3/3 | 5.57x 10^3^ | 1.97 | | 3/3 | | 5.46x 10^3^ | 1.47 | | 111.53 |
| Bsh-ha-2.0 | 3/3 | 5.73x 10^3^ | 4.09 | 3/3 | 5.39x 10^2^ | 4.39 | | 3/3 | | 5.49x 10^2^ | 2.42 | | 110.78 |
| Bsh-ha-1.0 | 3/3 | 5.40x 10^2^ | 22.23 | 3/3 | 5.50x 10^1^ | 13.40 | | 3/3 | | 5.02x 10^1^ | 12.94 | | 106.18 |
| Bsh-ha-0.5 | 3/3 | 5.45x 10^0^ | **40.78** | 3/3 | 1.52x 10^0^ | **40.57** | | 3/3 | | 1.77x 10^0^ | **48.36** | | **58.32** |
| Bsh-ha-0.25 | 1/3 | 3.68x 10^0^ | **57.74** | 3/3 | 2.08x 10^0^ | 24.96 | | 2/3 | | 3.97x 10^0^ | **58.37** | | **129.69** |
| Bsh-ha-0.1 | 2/3 | 1.41x 10^0^ | **60.19** | 1/3 | 8.99x 10^0^ | **57.74** | | 1/3 | | 6.00x 10^-1^ | **57.74** | | **193.93** |

Det = Detected,

Bold font indicated results outside range of acceptance criteria.

*N=1, ** N=2,

**Supplemental Table 6:** **Stability of the HA Standard**

| Days | N | Daily Mean Efficiency values | %CV |
| --- | --- | --- | --- |
| 0 | 3 | 1.957 | 1.067 |
| 1 | 5 | 1.929 | 1.128 |
| 2 | 7 | 1.952 | 0.483 |
| 5 | 3 | 1.958 | 0.771 |
| 6 | 3 | 1.952 | 0.237 |
| 7 | 5 | 1.947 | 0.547 |
| 8 | 3 | 1.950 | 0.320 |
| 9 | 4 | 1.954 | 0.975 |
| 12 | 1 | 1.919 | NA |
| 13 | 3 | 1.938 | 0.947 |
| 15 | 6 | 1.943 | 0.550 |
| 16 | 3 | 1.932 | 0.467 |
| 17 | 4 | 1.921 | 0.611 |
| 21 | 4 | 1.983 | 1.032 |
| 22 | 3 | 1.911 | 3.433 |
| 23 | 3 | 1.980 | 0.127 |
| 27 | 6 | 1.956 | 1.659 |
| 28 | 2 | 1.957 | 1.936 |
| 36 | 2 | 1.957 | 1.951 |
| 37 | 4 | 1.958 | 2.839 |
| 38 | 5 | 1.941 | 2.209 |
| 90 | 3 | 1.991 | 0.050 |
| 91 | 2 | 1.998 | 0.212 |
| **Total** 84  **Mean Efficiency**  **Overall %CV** | | 1.952 | 1.1 |

**Supplemental Table 7:** **Stability testing of HA Standard, PEC, and MGB Master Mix**

|  | %CV | | |
| --- | --- | --- | --- |
|  | Freeze thawed | PEC Lots #1 and #2 | MGB Lots A and B |
| Test Samples | (N=3) | (N=4) | (N=4) |
| 10^6^ STD | 4.52 | NA | 12.87 |
| 10^4^ STD | 1.94 | NA | 11.58 |
| 10^2^ STD | 11.58 | NA | 16.61 |
| PEC | 35.75 | 17.01 | NA |
| PTS (NCS + virus) | 20.86 | NA | NA |

NA = Not tested

**Supplemental Table 8:** **Specificity testing using different viral DNA**

| Virus | GC/ 5 μl |
| --- | --- |
| HSV-1 | Not detected |
| HSV-1 diluted | Not detected |
| HSV-2 | **5.26** |
| HSV-2 diluted | Not detected |
| Camelpox | 3.90x 10^4^ |
| Camelpox diluted | 3.12x 10^2^ |
| Vaccinia | 1.65x 10^3^ |
| Vaccinia diluted | 1.15x 10^1^ |
| Rabbitpox | 6.83x 10^2^ |
| Rabbitpox diluted | 6.84x 10^0^ |
| Cowpox | 2.59x 10^3^ |
| Cowpox diluted | 2.71x 10^1^ |

Bold font indicated results outside range of acceptance criteria

**Supplemental Table 9:** **Ruggedness testing of MGB Master Mix**

|  | | %CV | |
| --- | --- | --- | --- |
| Samples Tested | N | Assay #1 | Assay #2 |
| Standard 5x 10^6^ | 4 | 2.22 | 1.54 |
| Standard 5x 10^5^ | 4 | 0.60 | 2.34 |
| Standard 5x 10^4^ | 4 | 2.70 | 1.32 |
| Standard 5x 10^3^ | 4 | 4.50 | 1.74 |
| Standard 5x 10^2^ | 4 | 3.50 | 1.71 |
| Standard 5x 10^1^ | 4 | 23.39 | 13.87 |
| PEC | 4 | **47.47** | 22.78 |
| PEC aliquot A | 4 | 38.59 | 24.60 |
| PEC aliquot B | 4 | 39.86 | 23.60 |
| PEC aliquot C | 4 | 37.61 | 24.79 |

Bold font indicated results outside range of acceptance criteria.

**Supplemental Table 10:** **Ruggedness testing of extraction kits**

|  | Extraction kit lots: | |
| --- | --- | --- |
|  | Lot#1 | Lot#2 |
| Sample | GC/5ul | GC/5ul |
| PEC | 1.08x 10^4^ | NA |
| PEC | 1.15x 10^4^ | NA |
| Aliquot A | 1.60x 10^4^ | NA |
| Aliquot A | 1.63x 10^4^ | NA |
| Aliquot B | 4.07x 10^4^*(dropped) | NA |
| Aliquot B | 4.21x 10^4^*(dropped) | NA |
| Aliquot C | 1.25x 10^4^ | NA |
| Aliquot C | 1.20x 10^4^ | NA |
| PEC | NA | 1.35x 10^4^ |
| PEC | NA | 1.33x 10^4^ |
| Aliquot D | NA | 1.49x 10^4^ |
| Aliquot D | NA | 1.52x 10^4^ |
| Aliquot E | NA | 1.40x 10^4^ |
| Aliquot E | NA | 1.40x 10^4^ |
| Aliquot F | NA | 1.67x 10^4^ |
| Aliquot F | NA | 1.64x 10^4^ |
| Mean | 1.32x 10^4^ | 1.48x 10^4^ |
| SD | 2.37x10^3^ | 1.28x10^3^ |
| %CV | 17.98 | 8.70 |
| Mean | 1.43x 10^4^ | |
| SD | 1.92x10^3^ | |
| %CV | 13.41 | |

* Dixon’s Gap Test Where N is less than 9

**Supplemental Table 11: Ruggedness testing using two LightCyclers instruments**

|  | **Runs:** | |
| --- | --- | --- |
| Sample | VP42  VP44  %CV | VP43  VP45  %CV |
| Standard 5x 10^6^ | 2.13 | 1.06 |
| Standard 5x 10^5^ | 1.14 | 2.20 |
| Standard 5x 10^4^ | 2.63 | 1.04 |
| Standard 5x 10^3^ | 4.06 | 2.29 |
| Standard 5x 10^2^ | 3.39 | 2.47 |
| Standard 5x 10^1^ | 7.75 | 25.56 |
| Positive Control | 22.23 | 5.21 |
| PEC sample A | 10.99 | 4.22 |
| PEC sample B | 11.30 | 7.47 |
| PEC sample C | 10.51 | 3.44 |
